# Protein Interactions of the Mechanosensory Proteins Wsc2 and Wsc3 for Stress Resistance in *Saccharomyces cerevisiae*

**DOI:** 10.1534/g3.120.401468

**Published:** 2020-07-08

**Authors:** Vladimir Vélez-Segarra, Sahily González-Crespo, Ednalise Santiago-Cartagena, Luis E. Vázquez-Quiñones, Nelson Martínez-Matías, Yamirelis Otero, Julián J. Zayas, Rafael Siaca, Jeanmadi del Rosario, Inoushka Mejías, José J. Aponte, Noelani C. Collazo, Francisco J. Lasso, Jamie Snider, Matthew Jessulat, Hiroyuki Aoki, Brian C. Rymond, Mohan Babu, Igor Stagljar, José R. Rodríguez-Medina

**Affiliations:** * Department of Biochemistry, University of Puerto Rico, Medical Sciences Campus, PO Box 365067, San Juan, PR 00936-067; † School of Science and Technology, University Ana G. Mendez, Cupey Campus, Ana G Mendez Ave, No.1399, San Juan, PR 00926; ‡ Donnelly Centre, Department of Biochemistry, and Department of Molecular Genetics, University of Toronto, Ontario M5S 3E1, Canada; § Department of Biochemistry, University of Regina, Regina, Saskatchewan S4S 0A2, Canada; ** Department of Biology, University of Kentucky, Lexington, KY 40506; †† Mediterranean Institute for Life Sciences, Split, Croatia

**Keywords:** mechanosensory proteins, cell wall integrity pathway, stress response, protein-protein interactions

## Abstract

Antifungal drug discovery and design is very challenging because of the considerable similarities in genetic features and metabolic pathways between fungi and humans. However, cell wall composition represents a notable point of divergence. Therefore, a research strategy was designed to improve our understanding of the mechanisms for maintaining fungal cell wall integrity, and to identify potential targets for new drugs that modulate the underlying protein-protein interactions in *Saccharomyces cerevisiae*. This study defines roles for *Wsc2p* and *Wsc3p* and their interacting protein partners in the cell wall integrity signaling and cell survival mechanisms that respond to treatments with fluconazole and hydrogen peroxide. By combined genetic and biochemical approaches, we report the discovery of 12 novel protein interactors of *Wsc2p* and *Wsc3p*. Of these, *Wsc2p* interacting partners *Gtt1p* and *Yck2p*, have opposing roles in the resistance and sensitivity to fluconazole treatments respectively. The interaction of *Wsc2p* with *Ras2p* was confirmed by iMYTH and IP-MS approaches and is shown to play a dominant role in response to oxidative stress induced by hydrogen peroxide. Consistent with an earlier study, *Ras2p* was also identified as an interacting partner of *Wsc1p* and *Mid2p* cell wall integrity signaling proteins. Collectively, this study expands the interaction networks of the mechanosensory proteins of the Cell Wall Integrity pathway.

The frequency of systemic infections caused by opportunistic fungal pathogens of the normal endogenous flora is increasing worldwide (Van Thiel *et al.* 2012; Boral *et al.* 2018). *Candida auris* is rapidly emerging as a cause of hospital-acquired multidrug-resistant fungal infections at a global level (Chowdhary *et al.* 2017), while drug-resistant strains of *Candida glabrata* are also becoming increasingly responsible for systemic infections of immunocompromised patients (Rosenwald *et al.* 2016). Similarly, fungal infections with *Aspergillus fumigatus* and *Cryptococcus neoformans* can be acquired from non–endogenous host surroundings (Badiee and Hashemizadeh 2014). Infections in patients with severe immunological impairment can have fatal consequences due to the development of resistance by these fungi to the limited number of therapeutic antifungal drugs currently available (Roemer and Krysan 2014; Sanglard 2016). Therefore, there is an urgent need for new therapeutic strategies to address drug resistance in fungal infections.

Multiple forms of environmental stresses that compromise cell wall structure or function can activate the MAPK cascade underpinning the Cell Wall Integrity (CWI) pathway. The initial steps for activating the CWI response occur through interactions mediated by the N-terminal regions of the *Wsc1p*, *Wsc2p*, *Wsc3*, *Mid2p* and *Mtl1p* mechanosensory proteins with the cell wall (Straede and Heinisch 2007; Heinisch *et al.* 2010; Kock *et al.* 2016). The binding of the GDP-GTP Exchange Factor (GEF), *Rom2p*, to the cytoplasmic C-terminal ends of these proteins and the activation of the small GTPase *Rho1p* are integral steps in CWI pathway activation (Ozaki *et al.* 1996; Philip and Levin 2001). However, proteins acting upstream of *Rom2p* recruitment in this activation step have yet to be identified.

The MAPK cascade directed by the CWI pathway is regulated by protein kinase C1, *Pkc1p* (Levin 2005; Fuchs and Mylonakis 2009) and a stress inducible phosphatase, *Sdp1p* (Hahn and Thiele 2002). *Rom2p* binding to the *Wsc1p* C-terminal tail requires dephosphorylation of specific serine residues of *Wsc1p* within a serine-rich motif, specifically S319, S320, S322, S323, (Vay *et al.* 2004) but the specific enzymes that phosphorylate and dephosphorylate these residues and how they interact with *Rom2p* are uncertain. In addition to chemical or genetic perturbation of the cell membrane, the CWI pathway is stimulated by a wide variety of other stresses including nutrient starvation, hypo-osmotic shock, heat shock, and environmental conditions that perturb cell integrity functions (Kamada *et al.* 1995; Fuchs and Mylonakis 2009). Readouts of the CWI pathway include the enhanced transcription of genes encoding stress proteins and cell wall components, altered translational regulation, and the modulation of growth processes (Kamada *et al.* 1995; Gray *et al.* 1997; de Nobel *et al.* 2000; Zu *et al.* 2001; Rodríguez-Quiñones *et al.* 2008; Rodríguez-Quiñones and Rodríguez-Medina 2009; Rivera-Ruiz *et al.* 2010; Rodicio and Heinisch 2010; Pagán-Mercado *et al.* 2012; Yurko *et al.* 2017; Leskoske *et al.* 2018). The CWI pathway also controls genes involved in other biological processes such as regulation of cytoskeleton polarization (de Nobel *et al.* 2000), the activity of cell wall biosynthetic enzymes (Levin 2011), polarized cell growth (Zarzov *et al.* 1996), and the control of target of rapamycin complex 1 (TORC1) (Petkova *et al.* 2010) and TORC2 complexes (Leskoske *et al.* 2018).

In the budding yeast *Saccharomyces cerevisiae*, mechanosensory proteins represented by the Wsc-family (*Wsc1p*, *Wsc2p*, and *Wsc3p*) and the Mid-family (*Mid2p* and its homolog *Mtl1p*), are responsible for detecting and transmitting cell wall, nutritional, and environmental stress cues through the CWI pathway (Verna *et al.* 1997). These proteins share amino acid sequence identity of approximately 30% between the Wsc-family members and 50% between the Mid-family members. The Wsc-family proteins are distinguished by the presence of a cysteine-rich domain at the N-terminal region which is covalently attached to cell wall glycoproteins via glucan linkages. They also contain a signal peptide for ER uptake, a region rich in serines and threonines that are highly O-mannosylated, a single transmembrane domain, and a relatively short cytoplasmic C-terminal tail (Kock *et al.* 2015). Despite their structural similarities, the Wsc and Mid family proteins are reported to elicit different responses to stress stimuli. For example, previous works have shown that *Wsc1p* and *Mid2p* are the primary cell wall integrity sensors in the response to cell wall stress (Verna *et al.* 1997; Philip and Levin 2001; Santiago-Cartagena *et al.* 2019), while *Wsc2p*, *Wsc3p*, and *Mtl1p* respond less strongly to cell wall stress but are described to be important for heat stress (Verna *et al.* 1997) and oxidative stress (Petkova *et al.* 2010), respectively. Curiously, despite the important role of these mechanosensory proteins in regulating activation of the CWI pathway, MAPK activation is not strictly required for cell growth under every cell wall stress condition. Therefore, it is proposed that multiple, and possibly redundant, survival pathways become activated by different types of stresses (Santiago-Cartagena *et al.* 2019).

There is a high level of conservation of mechanosensory proteins of the CWI pathway among fungal pathogens. Putative genes encoding homologs of the *S. cerevisiae* Wsc-family (AfWsc1-3) and Mid2-family (AfMidA) were previously identified in *Aspergillus fumigatus* by BLAST analysis (Dichtl *et al.* 2012; Dichtl *et al.* 2016). In *A. fumigatus*, mutants of components of the CWI pathway show the same sensitivity to compounds typically used to characterize the CWI pathway in *S. cerevisiae* (Valiante *et al.* 2015). The *Candida albicans* CWI pathway is activated by stresses similarly to *S. cerevisiae*, with *C. albicans* encoding CaWsc1p and CaWsc2p homologous to *Wsc1p* and *Wsc2p* of *S. cerevisiae*, respectively (Dichtl *et al.* 2012). The *Cryptococcus neoformans* MAPK module is composed of three members known as MAPKKK CnBck1, MAPKK CnMkk2, and MAPK CnMpk1 which are similar to *Bck1p*, *Mkk2p* and *Mpk1p* of *S. cerevisiae*, respectively. In humans, these proteins are non-existent or have evolved different functions making them potentially good therapeutic targets for antifungal agents. Due to the high level of homology among mechanosensory proteins and the components of MAPK module, it has become evident that other fungi including human pathogens share a CWI signal transduction mechanism similar to *S. cerevisiae*.

*Wsc2p* and *Wsc3p* are reported to have redundant functions. A *wsc2**Δ* null mutant strain exhibits reduced competitive fitness, consistent with a basic housekeeping function in cell biology (Wilk *et al.* 2010). A *wsc3**Δ* null mutant strain was reported to show increased sensitivity to arsenate, a response that was suppressed by sorbitol, suggesting that *Wsc3p* responds to cell wall stress treatments (Matia-González and Rodríguez-Gabriel 2011). Compared with a wildtype control, caspofungin treatment of *wsc2**∆* and *wsc3**∆* strains induces the increased accumulation of hyper-phosphorylated *Slt2p*, the readout for the CWI pathway activation (Reinoso-Martín *et al.* 2003). Double and triple mutants of *wsc1**∆* combined with *wsc2**∆*, *wsc3**∆*, or *wsc2**∆**wsc3**∆* mutation have been evaluated for growth phenotypes by Verna *et al.* (1997). Under normal growth conditions on synthetic defined (SC) medium at 28°, none of the single mutants *wsc1**∆*, *wsc2**∆*, or *wsc3**∆* were thermosensitive, while specific combinations of these mutants (*i.e.*, *wsc1**Δ**wsc2**Δ*, *wsc1**Δ**wsc3**Δ*, and *wsc1**Δ**wsc2**Δ**wsc3**Δ*) acquired the thermosensitive phenotype, supporting the notion that the *WSC* genes share redundant functions.

In a previously published study from our laboratory, we reported novel protein-protein interactions (PPIs) of *Wsc1p* and *Mid2p* (Santiago-Cartagena *et al.* 2019). Here, we aim to expand the known interactome map of *Wsc2p* and *Wsc3p* by identifying novel PPIs using the integrated Membrane Yeast Two Hybrid (iMYTH) screening method (Fetchko *et al.* 2003; Fetchko and Stagljar 2004; Paumi *et al.* 2007; Snider *et al.* 2010) and to score the newly identified factors for function in the regulation of growth and the activation of the CWI pathway in response to treatments with caspofungin (CS), fluconazole (FCZ), and hydrogen peroxide (H_2_O_2_). The results reported here provide new insight into the CWI signal transduction mechanism active in *S. cerevisiae* and other fungi.

## Materials and Methods

### Strains, transformations and growth conditions

To create iMYTH bait strains, iMYTH L2 or L3 cassettes containing the 35-40 bases of homology near the C-terminal end of the *WSC2* or *WSC3* gene (minus the stop codon), C-terminal fragment of ubiquitin, yellow fluorescence protein (L3 cassette only) and transcription factor [C_ub_-(YFP)-LexA-VP16 KanMX] were amplified by PCR. The resulting hybrid genes were transformed into MYTH reporter strains (THY.AP4 or L40) (Snider *et al.* 2010) and selected on YPAD solid medium containing G418 (200 μg/ml). The correct genomic construct of the bait strain was verified by PCR using a forward primer internal to the *WSC2* or *WSC3* genes and a reverse primer internal to the KanMX gene. The iMYTH bait validation and localization assays were performed as described (Snider *et al.* 2010; Santiago *et al.* 2016; Santiago-Cartagena *et al.* 2019).

The *wsc2**∆*, *wsc3**∆*, and prey protein single deletion mutant strains were generated by single gene replacement with a KanMX4 module by homologous recombination using a PCR based strategy (Wach *et al.* 1994) in the BY4742 genetic background (Open Biosystems). The wild type strains, BY4741 and BY4742, were obtained from ATCC. See Supplementary Table S1 for yeast strains and Supplementary Table S2 for DNA primers used in this study.

### Library screening

iMYTH library screens were conducted as described by Snider *et al.* 2010. Putative positive transformants obtained with 40-80 μg of N_ub_G-X cDNA prey library were selected on BD-Trays (Beckton-Dickinson) containing Synthetic Dropout medium without Tryptophan, Adenine, and Histidine (SD-WAH) or Synthetic Dropout medium without Tryptophan and Histidine (SD-WH) incubated at 30° for 3-4 days to select for markers *TRP1* present on the vector DNA, or *ADE2* and *HIS3* that are integrated into the genome as part of the iMYTH reporter system. For the Optimized Large Scale Transformation, 20 ml of bait strain culture in 2X Yeast Peptone hydrolysate plus Dextrose broth medium (YPD) was transformed with 40-80 μg of N_ub_G-X cDNA prey library.

### Plasmid recovery and sequencing

Single colonies obtained from the large-scale iMYTH transformation of bait strains were picked and diluted into 50 μl sterile 0.9% NaCl solution. Afterward, 2.5 μl of re-suspended cells were plated onto SD-WAH or SD-WH plates containing X-Gal and grown for 1-3 days at 30° to screen for *trans*-activation of the lacZ reporter gene. Blue colonies were inoculated into SD-W in 96-well blocks and grown for 2 days at 30°. The pellets were re-suspended with 125 μl of Lysis solution [β-mercaptoethanol, Solution A (1M Sorbitol, 0.1 M Sodium Citrate, 60 mM EDTA) and Zymolase Solution (Zymolase powder, 1 M Sorbitol)] and treated for 2 hr at 37°. Plasmid DNA from these transformants was isolated using Nucleospin 96-well miniprep kits were according to the manufacturer’s protocol.

Competent DH5α *E. coli* was transformed with the plasmids recovered from the yeast minipreps and plated on LB agar with 100 μg/ml ampicillin. For high-throughput transformation, 96-well plates and 96-well blocks were used. For *E. coli* minipreps, single colonies were inoculated in Terrific Broth containing 100 μg/ml ampicillin and grown for 2 days at 37°. The recovered plasmids were purified using Nucleospin columns and the plasmid DNA sequenced using the N_ub_G forward internal primer (Table S2). In-house software (developed at the University of Toronto) was used for large-scale BLAST analysis and identification of yeast protein sequences.

In the primary screen, a total of 885 colonies for *Wsc2p* and 406 colonies for *Wsc3p* containing cDNAs encoding putative interactors were picked from the transformation media. From 233 colonies for *Wsc2p* and 178 colonies for *Wsc3p*, 280 cultures yielded plasmid DNA that could be re-screened for activity. 259 cDNA clones that were identified by Sanger sequencing represented 58 different proteins that underwent Bait Dependency Tests. 15 out the 58 cDNAs passed the Bait Dependency Tests.

### Bait dependency test

Bait Dependency Tests (BDT) were performed to validate iMYTH positive clones (Snider *et al.* 2010). The purified prey plasmids were transformed into the yeast bait strains *Wsc2p* THY L2, *Wsc3p* THY L3, and A0286 or *Wsc2p* L40 L3, *Wsc3p* L40 L3 and A0287. Note that A0286 and A0287 correspond to ‘negative control’ reporter strains stably expressing artificial bait construct, in THY.AP4 and L40 background, respectively. The resulting transformations were plated onto SD-W media and incubated at 30° for 3-4 days. The transformant colonies were selected in triplicate and plated onto SD-WAH + X-Gal or SD-WH + X-Gal. Preys that caused growth and blue coloration in a bait strain, but not in control bait strain, were considered to be specific or true interactors (Paumi *et al.* 2007; Snider *et al.* 2010). The specific interactors for the mechanosensory proteins were classified according to their biological process derived from SGD (Cherry *et al.,* 2012). The Cytoscape, version 3.2.1, software (Shannon *et al.* 2003) was used to generate protein interactome maps.

### Western blot assay for activation of the CWI pathway under stress conditions

To induce cell wall stress, 75 ng/ml of CS was added to 25 ml of culture at OD_600_ ∼0.7-0.9. For induction of oxidative stress, hydrogen peroxide (H_2_O_2_) was added at a final concentration of 1 mM to 25 ml of culture at OD_600_ ∼0.7-0.9. Plasma membrane stress was induced with 100 µM FCZ added to cultures under similar conditions. All cultures were incubated at 27° for 1 hr before harvesting. In all subsequent assays, the results obtained with the experimental cultures were compared to yeast cultures without any stress treatment.

For protein recovery, yeast cultures (25 ml) were centrifuged for 5 min at 3,838 × g and then transferred to 1.5 ml microcentrifuge tubes. The cells were washed in ice cold distilled water, centrifuged at 17,530 × g for 3 min at 4° and pellets suspended in 0.5 ml lysis buffer (50 mM Tris-HCl, pH 7.5, 10% glycerol, 1% TritonX-100, 0.1% SDS, 150 mM NaCl, 5 mM EDTA) supplemented with 5X Protease Inhibitor Cocktail (PIC) (Roche), 1X phosphatase inhibitor cocktail (II and III, Sigma) and 5X PMSF (10 mM). The cells were mechanically disrupted by the addition of approximately 200 µl of sterile glass beads followed by five sets of alternate vortexing at full speed for 45 sec followed by 3 min incubation on ice. After disruption, the contents in the tubes were centrifuged at 17,530 × g for 10 min at 4° and the clarified protein supernatants transferred to new pre-chilled 1.5 ml microtubes. An aliquot of each extract was used for determining protein concentration using the DC Protein Assay (Bio-Rad). Afterward, 50 µg of total protein was denatured by heating at 95° in the presence of a 4X Laemmli dye solution and 5% per volume β-mercaptoethanol for 10 min and then separated in 10% polyacrylamide gels by SDS-PAGE. The proteins in the gels were then transferred to nitrocellulose membranes (BioRad) using a constant electrical current for 90 min at 4°. The membranes were probed with the following primary antibodies: anti-human phospho-p44/42 MAPK rabbit monoclonal (Cell Signaling Technology) and yeast Phosphoglycerate kinase (*Pgk1p*) mouse monoclonal (Molecular Probes, Invitrogen) at a 1:1,000 dilution. The primary antibodies were diluted in Odyssey blocking buffer (LI-COR) and incubated at room temperature (RT) for 1 hr. The membranes were washed two times with 1X PBS/ 0.2% Tween20 for 10 min per wash. The secondary antibodies used were Goat anti-Rabbit IRDye 800CW (1:10,000) LI-COR (green) and Goat anti-Mouse IRDye 680LT (1:10,000) LI-COR (red). The membranes were incubated with secondary antibodies for 30 min at RT and washed as described above. 1X PBS was used to wash the membranes before scanning with an Odyssey CLx Infrared Fluorescent Imaging System (LI-COR). Numerical values expressed in Figures 5A-5C represented as the fold-change of the P-*Slt2p* band intensity in each experimental sample, adjusted to the P-*Slt2p* band intensity of wild type control sample (WT), each normalized against the intensity of *Pgk1p*. For statistical analysis by the Student T Test, a minimum of 3 replicates were conducted per experiment and each data point shown represents the mean ± SEM of n ≥ 3.

### Growth analysis

Growth analysis by a spotting test (Kwolek-Mirek and Zadrag-Tecza 2014) was performed using 1 × 10^8^ cell/ml aliquots (determined by direct cell count of culture dilutions) taken from cultures at OD_600_ between 0.5-0.8. Strains bearing deletion mutations of the genes encoding bait proteins, their corresponding newly identified protein partners, and double-mutant strains were compared with wild type strain BY4742 controls (Table S1). For agar plate assays, 3 μl drops taken from 1/10 serial dilutions of the working culture (ranging from 10^8^ to 10^2^ cell/ml) were inoculated in triplicate on CSM agar plates under the following conditions: normal conditions (30°, no treatment), oxidative stress (1 mM H_2_O_2_), cell wall stress (75 ng/ml CS) and plasma membrane stress (100 µM FCZ). Plates were incubated at 30° for 2-3 days. Relative growth on agar plates was quantified by comparing the density of growth within each drop displayed by the wild type strain across all dilutions, to the density of growth displayed by the mutant strains across the same dilution series. Relative growth measurements were classified as follows: density of growth equivalent to a wild type strain was described as “No Phenotype” (NP), greater than the wild type by one or more serial dilutions was described as “Resistant” (R), less than the wild type by one or more serial dilutions was described as “Sensitive” (S) (Figures 4A-4C and Tables S3-S6). Strains that exhibited the (S) phenotype in the spotting tests were subjected to corroboration by the Colony Forming Units (CFU) assay (Table S7) (Kwolek-Mirek and Zadrag-Tecza 2014).

### Immunoprecipitation coupled with Mass Spectrometry (IP-MS)

Five (5) ml cultures containing *Wsc2p*-GFP, *Wsc3p*-GFP and BY4742 strains were grown in YPD at 30° overnight. 25 ml yeast cultures in YPD media were inoculated with these overnight cultures and grown to an OD_600_∼1.0 for subsequent assessment of bait expression by western blot. The remaining seed culture was used for inoculation of a 1L culture and incubated overnight. Subsequently, the 1L cultures were centrifuged in 250ml bottles at 2,041 × g for 15 min at 4°. The pellets were resuspended with 2.5 ml of IPLB (20 mM Hepes KOH, pH 7.4, 150 mM KOAc, 2 mM Mg(Ac)_2_, 1 mM EGTA, 10% glycerol and 1X PIC (Protease Inhibitor Cocktail) and lysed mechanically as described previously. For protein crosslinking reaction, 2.5 ml of clarified lysate containing approximately 800 µg total protein was mixed with 1 mM Dithiobis (Succinimidyl Propionate) (DSP) and incubated on ice for 30 min with gentle inversion every 10 min. After incubation, 100 mM of 1 M Tris-HCl pH 7.5 was added and incubated on ice for 10 min with gentle inversion every 5 min. A solution of 1% Digitonin was added to the reaction and incubated on ice for 30 min. The samples were then clarified by centrifugation at 2,665 × g for 15 min at 4°. An aliquot of 50 μl of μMACS Anti-HA Microbeads (Miltenyi Biotec) were added to 1.25 ml of supernatant (∼400 µg total protein) to capture TAP tagged bait-prey complexes and incubated on ice for 30 min. The MACS columns (Miltenyi Biotec) in a magnetic holder were equilibrated with 250 μl of IPLB + 1% digitonin + 1X PIC. The supernatants were added to the columns and for the wash step the columns were washed with 1 ml IPLB + 0.1% digitonin + 1X PIC two times and one time with 1 ml IPLB. For the on-bead trypsin digestion, 25 μl of EB I (2 M Urea, 50 mM Tris-HCl, pH 7.5, 25 mM DTT and 5 μg/ml trypsin) was added and incubated for 30 min at RT. 100 μl of EB II (2 M Urea, 50 mM Tris-HCl, pH 7.5 and 5 mM chloroacetamide) was added to the column and the eluted fractions were collected. The digestion was continued overnight at RT and 1 μl of 100% TFA was added to stop the reaction. The sample was cleaned and desalted as previously described (Santiago-Cartagena *et al.* 2019), except that the peptides were eluted using 1% acetic acid, 65% acetonitrile solution. The peptides were dried by evaporation and dissolved with 1% formic acid. Each sample was subjected to analysis with an Orbitrap Elite mass spectrometer (Thermo Fisher Scientific) to generate MS/MS spectra. The results obtained were matched against a yeast protein sequence database using SEQUEST search engine and scored according to the number of unique peptides identified and the STATQUEST algorithm. Matches were considered valid if they contained two or more unique peptide fragments with matches of ≥95% probability.

### Bioinformatics analysis

Protein sequences with homology to *Wsc2p* and *Wsc3p* and the 12 iMYTH interactors that could be retrieved by conducting a comprehensive homology search in the public biological databases from The UniProt Consortium (2017) and European Molecular Biology Laboratory - European Bioinformatics Institute (EMBL-EBI) with Multiple Sequence Alignment Programs were obtained by using specific Structure Query Language (SQL) queries (Nicholas *et al.* 2000). Bioinformatics analysis was performed using the application Multiple Sequence Comparison by Log-Expectation (MUSCLE) (Edgar 2004) (Table 3). The acquired data were evaluated using two criteria: The first criterion was selection of the proteins that were conserved in yeast *Saccharomyces cerevisiae*, *Aspergillus fumigatus*, *Candida albicans*, *Cryptococcus neoformans*, and *Homo sapiens*. This selection was performed by a series of specific queries in SQL using the following names and alternative names for all proteins excluding *Gtt1p*, *Zeo1p*, and *Wsc1p* that were not conserved in *H. sapiens*. The first criterion was met by proteins: *Bmh1p*, *Cpr1p*, *Egd1p*, *Egd2p*, *Msa1p*, *Ras2p*, *Tma7p*, *Yck1p*, *Yck2p*, Ypl199cp, *Mid2*, *Mtl1p*, *Wsc2p*, and *Wsc3p*. The second criterion was inclusion of all proteins (interactors and mechanosensory proteins) that shared homology with *Saccharomyces cerevisiae* and at least one of the following fungi: *Aspergillus fumigatus*, *Candida albicans*, or *Cryptococcus neoformans*, and did not share any homology with *Homo sapiens*.

### Data availability

All strains and reagents are available upon request to the corresponding author. Supplemental Materials: Table S1. Double mutant strains used in this study; Table S2. Primers used in this study; Table S3. Susceptibility profiles of single mutant strains of *Wsc2p* interactors; Table S4. Susceptibility profiles of double mutant strains of *Wsc2p* and interactors; Table S5. Susceptibility profiles of single mutant strains of *Wsc3p* interactors; Table S6. Susceptibility profiles of double mutant strains of *Wsc3p* and interactors; Table S7. Colony Forming Units (CFU) assay of hypersensitive mutant strains of *Wsc2p*, *Wsc3p*, and their interactors; Figure S1. A representative spotting test for growth of null mutant strains of *Wsc2p*, *Wsc3p*, and their interactors treated with 75 ng/ml CS; Figure S2. Western blot analysis of phospho-*Slt2p* (P-*Slt2p*) levels in null mutant strains of *Wsc2p* and *Yck2p* treated with 75 ng/ml CS. Supplemental material available at figshare: https://doi.org/10.25387/g3.12453707.

## Results

### Confirmation of expression and localization of Wsc2p and Wsc3p iMYTH bait constructs

**Figure 1:**
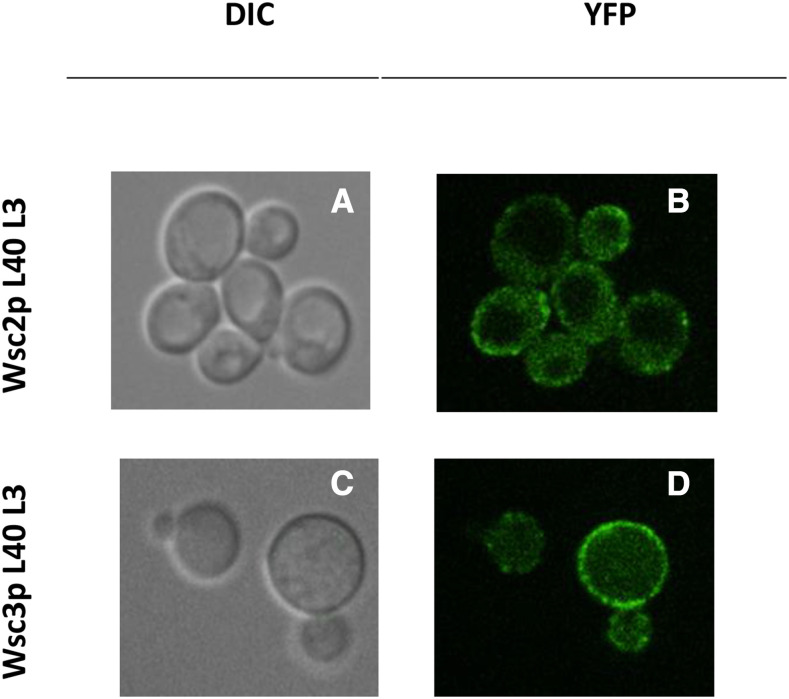
Representative DIC and YFP images for the localization of *Wsc2p* and *Wsc3p* in the plasma membrane. A) and C) display DIC images for *Wsc2p* L40 L3 and *Wsc3p* L40 L3 strains respectively. B) and D) show fluorescent images of *Wsc2p* L40 L3 and *Wsc3p* L40 L3 strains respectively. Images were acquired with a Leica DMI 6000B Inverted Confocal Microscope with a field size of 16 µm. YFP = Yellow Fluorescent Protein, DIC = Differential Interference Contrast.

**Figure 2:**
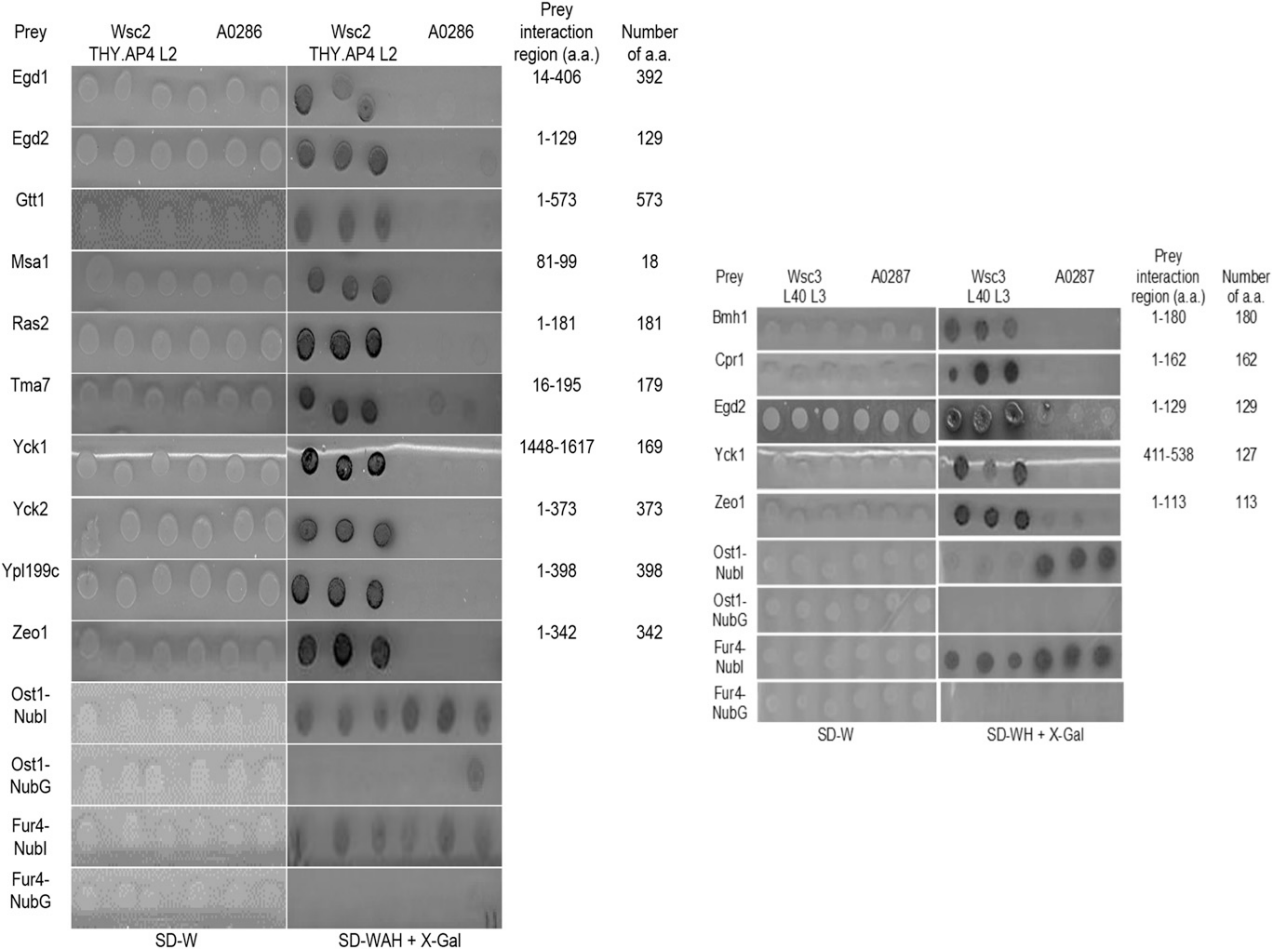
Bait Dependency Testing of *Wsc2p* and *Wsc3p* interactor proteins identified by iMYTH. Yeast cells expressing *Wsc2p*-C_ub_-TF and *Wsc3p*-C_ub_-TF were transformed with preliminary interactors and N_ub_G/N_ub_I control plasmids. Transformants were verified on *SD-W* medium. Interaction between the bait and prey was demonstrated by growth of the *Wsc2p*-C_ub_-TF THY.AP4 L2 (used in this screen because the results of the self activation tests were visualized more clearly in this strain) and absence of growth by strain A0286 on *SD-WAH* + *X-Gal* media, or *Wsc3p*-C_ub_-TF L40 L3 and absence of growth by strain A0287 on *SD-WH + X-Gal* media. Transformants were spotted in triplicate. Left panel contains: *Wsc2p*-C_ub_-TF in THY.AP4 L2 background. Right panel contains: *Wsc3p*-C_ub_-TF in L40 L3 background. Three replicates were done for each experiment.

An iMYTH assay was conducted to detect protein-protein interactions of the mechanosensors *Wsc2p* and *Wsc3p*. *Wsc2p* and *Wsc3p* prey proteins were used to identify cytoplasmic and membrane-associated prey proteins *in vivo* as described previously (Snider *et al.* 2010; Santiago-Cartagena *et al.* 2019). Fluorescence microscopy (Figure 1) was used to demonstrate that both the *Wsc2p* and *Wsc3p* bait proteins were expressed and correctly localized to the plasma membrane. The clustered appearance of the *Wsc2p*-YFP and *Wsc3p*-YFP fluorescence at the cell periphery was reminiscent of the pattern previously observed for *Mid2p*-YFP (Santiago-Cartagena *et al.* 2019). The *Wsc2p* and *Wsc3p* bait proteins did not self-activate in the absence of interacting prey using the N_ub_I/N_ub_G control tests (Figure 2) (Snider *et al.* 2010).

### Protein interaction network map of Wsc2p and Wsc3p

**Figure 3:**
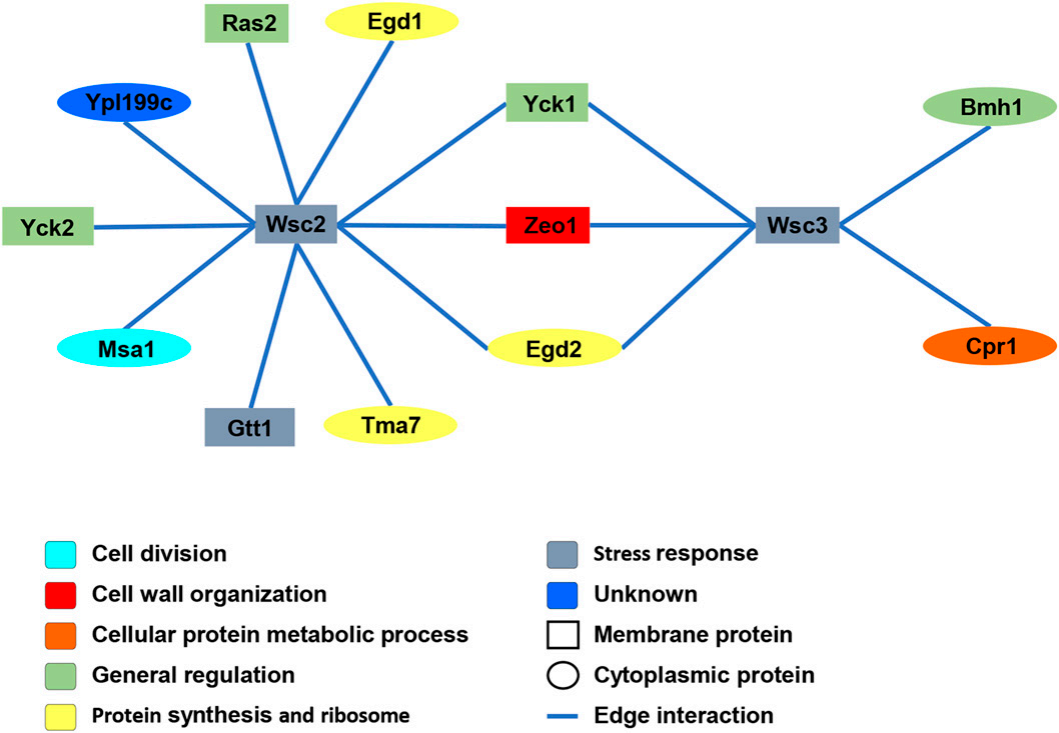
Interactome map of *Wsc2p* and *Wsc3p* interactor proteins identified by iMYTH and validated by Bait Dependency Tests (BDT). Geometric shapes of the nodes represent the cellular localization of the proteins. *Wsc2p* and *Wsc3p* are the bait proteins and the surrounding nodes represent prey proteins. Node fill color-code indicates the biological process for each protein. All edges indicate a physical protein-protein interaction.

Prey proteins that interacted with the *Wsc2p* and *Wsc3p* baits were identified in selection media, and the corresponding plasmids recovered and cDNAs sequenced. Fifteen out of 58 different proteins identified (26%) were confirmed by Bait Dependency Tests (BDTs) (Snider *et al.* 2010) (Figure 2). These encoded 10 protein interactors for *Wsc2p* (*Egd1p*, *Egd2p*, *Gtt1p*, *Msa1p*, *Ras2p*, *Tma7p*, *Yck1p*, *Yck2p*, *Ypl199c*, *Zeo1p*) and 5 protein interactors for *Wsc3p* (*Bmh1p*, *Cpr1p*, *Egd2p*, *Yck1p*, and *Zeo1p*) (Figure 2 and Table 1) of which *Yck1p*, *Zeo1p*, and *Egd2p* were shared by *Wsc2p* and *Wsc3p* (Figure 3 and Table 1). Unique interactors were verified by cross-testing in each of the bait strains. With the exception of *Zeo1p*, these interactors were novel and not reported previously in the *Saccharomyces* Genome Database (yeastgenome.org) (Cherry *et al.,* 2012). A map of the interaction network for the 12 interactors of *Wsc2p* and *Wsc3p* identified by iMYTH and confirmed by BDT was generated (Figure 3). The SGD reports 20 additional physical interactors for *Wsc2p* and 13 for *Wsc3p*, identified by other methods, that were not identified in our iMYTH screen. The biological functions described in the SGD (yeastgenome.org) (Cherry *et al.,* 2012) for *Wsc2p*, *Wsc3p*, and their iMYTH protein interactors are listed in Table 1.

**Table 1:** Wsc2p and Wsc3p interacting proteins identified by iMYTH assay and confirmed by BDT

Gene Name	Systemic Name	Bait Gene	Description (According to SGD, http://www.yeastgenome.org/)
BMH1	YER177W	WSC3P	14-3-3 protein, major isoform; controls proteome at post-transcriptional level, binds proteins and DNA, involved in regulation of exocytosis, vesicle transport, Ras/MAPK and rapamycin-sensitive signaling, aggresome formation, spindle position checkpoint; protein increases in abundance and relative distribution to the nucleus increases upon DNA replication stress; antiapoptotic gene similar to human 14-3-3; BMH1 has a paralog, BMH2, that arose from whole genome duplication.
CPR1	YDR155C	WSC3P	Cytoplasmic peptidyl-prolyl *cis*-trans isomerase (cyclophilin); catalyzes the *cis*-trans isomerization of peptide bonds N-terminal to proline residues; binds the drug cyclosporin A; N-terminally propionylated *in vivo*; protein abundance increases in response to DNA replication stress.
EGD1	YPL037C	WSC2P	Subunit beta1 of the nascent polypeptide-associated complex (NAC); involved in protein targeting, associated with cytoplasmic ribosomes; enhances DNA binding of the Gal4p activator; homolog of human BTF3b; EGD1 has a paralog, BTT1, that arose from the whole genome duplication.
EGD2	YHR193C	WSC2P, WSC3P	Alpha subunit of the nascent polypeptide-associated complex (NAC); involved in protein sorting and translocation; associated with cytoplasmic ribosomes.
GTT1	YIR038C	WSC2P	ER associated glutathione S-transferase; capable of homodimerization; glutathione transferase for Yvc1p vacuolar cation channel; expression induced during the diauxic shift and throughout stationary phase; functional overlap with Gtt2p, Grx1p, and Grx2p.
MSA1	YOR066W	WSC2P	Activator of G1-specific transcription factors MBF and SBF; involved in regulation of the timing of G1-specific gene transcription and cell cycle initiation; localization is cell-cycle dependent and regulated by Cdc28p phosphorylation; MSA1 has a paralog, MSA2, that arose from the whole genome duplication.
RAS2	YNL098C	WSC2P	GTP-binding protein; regulates nitrogen starvation response, sporulation, and filamentous growth; farnesylation and palmitoylation required for activity and localization to plasma membrane; homolog of mammalian Ras proto-oncogenes; RAS2 has a paralog, RAS1, that arose from the whole genome duplication.
TMA7	YLR262C-A	WSC2P	Protein of unknown that associates with ribosomes; null mutant exhibits translation defects, altered polyribosome profiles, and resistance to the translation inhibitor anisomcyin; protein abundance increases in response to DNA replication stress.
YCK1	YHR135C	WSC2P, WSC3P	Palmitoylated plasma membrane-bound casein kinase I (CK1) isoform; shares redundant functions with Yck2p in morphogenesis, proper septin assembly, endocytic trafficking, and glucose sensing; stabilized by Sod1p binding in the presence of glucose and oxygen, causing glucose repression of respiratory metabolism; involved in the phosphorylation and regulation of glucose sensor Rgt2p; YCK1 has a paralog, YCK2, that arose from the whole genome duplication.
YCK2	YNL154C	WSC2P	Palmitoylated plasma membrane-bound casein kinase I (CK1) isoform; shares redundant functions with Yck1p in morphogenesis, proper septin assembly, endocytic trafficking, and glucose sensing; stabilized by Sod1p binding in the presence of glucose and oxygen, causing glucose repression of respiratory metabolism; involved in the phosphorylation and regulation of glucose sensor Rgt2p; YCK2 has a paralog, YCK1, that arose from the whole genome duplication.
YPL199C	YPL199C	WSC2P	Putative protein of unknown function; predicted to be palmitoylated.
ZEO1	YOL109W	WSC2P,WSC3P	Peripheral membrane protein of the plasma membrane; interacts with Mid2p; regulates the cell integrity pathway mediated by Pkc1p and Slt2p; the authentic protein is detected in a phosphorylated state in highly purified mitochondria.

### Assessment of Wsc2p and Wsc3p interactor function in resistance to antifungal treatments

**Figure 4:**
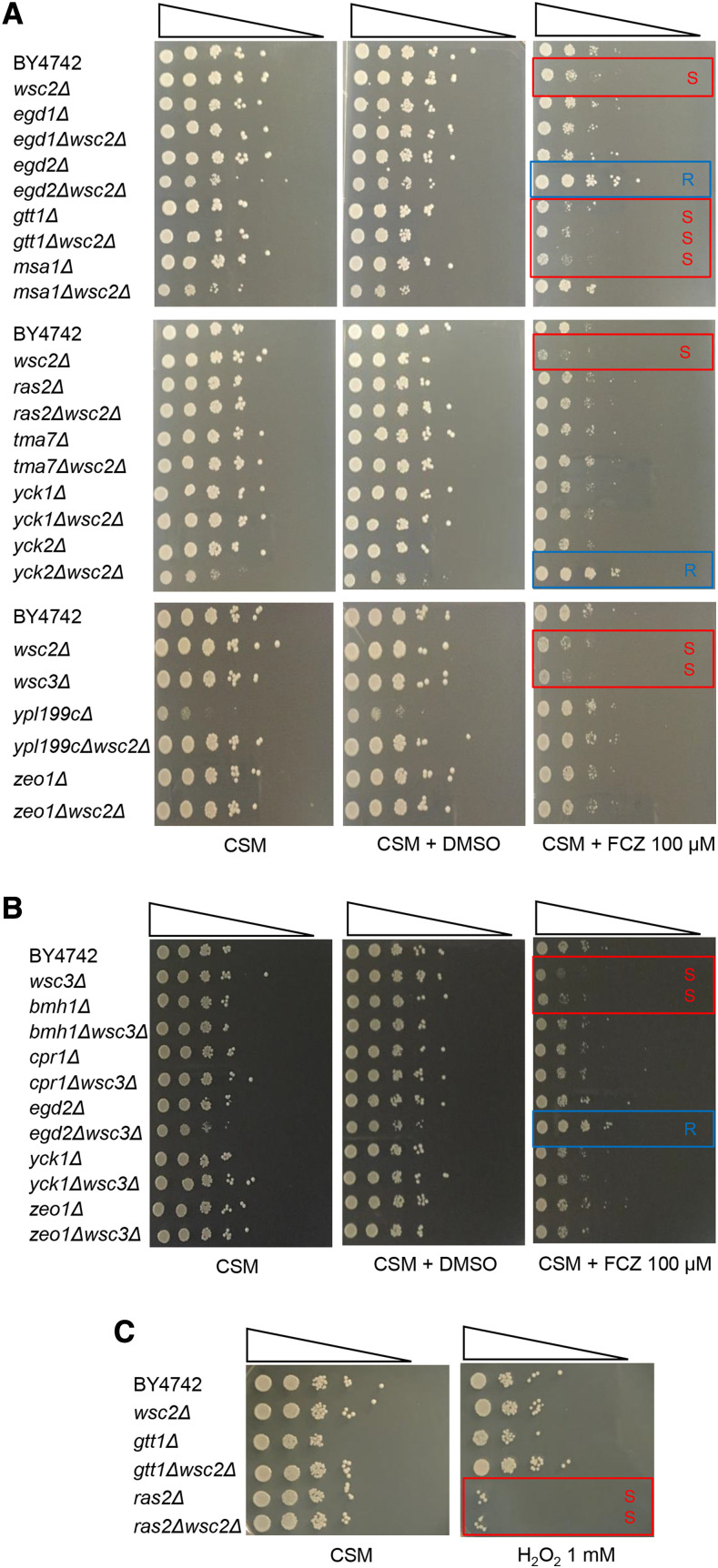
A representative spotting test of mutant strains of *Wsc2p* and *Wsc3p* and their interactors exposed to stress conditions. Identical volumes representing 10-fold serial dilutions (10^8^-10^2^) of wild type (BY4742α), single, and double mutants, were spotted onto CSM plates and incubated at 30°. From left to right, A: *wsc2**Δ* interactors with no treatment (CSM), treated with 2% DMSO vehicle, or treated with 100 μM Fluconazole (FCZ); B: *wsc3**Δ* interactors on CSM, treated with 2% DMSO vehicle, or treated with 100 μM FCZ; and C: *gtt1**Δ* and *ras2**Δ* strains on CSM, or treated with 1 mM hydrogen peroxide (H_2_O_2_). The plates were inspected after two days of incubation. Three replicates were done for each experiment.

We next evaluated the growth of yeast bearing single or double deletions of the genes defined by the iMYTH interactions. Assays were performed in the absence of drug and in the presence of the antifungals caspofungin (CS), an inhibitor of the fungal β-1,3-glucan synthetase, an essential component of the yeast cell wall, thereby disturbing the integrity of the fungal cell wall (Deresinski and Stevens 2003) and fluconazole (FCZ), which inhibits fungal ergosterol synthesis by inhibiting a P-450 enzyme (C-14 α-demethylase) required for the formation of ergosterol, an essential component of the fungal cytoplasmic membrane (Roemer and Krysan 2014). Drug sensitivity was defined as described in the Materials and Methods (Figures 4A and 4B).

To a first approximation, with the exception of the slow growing *ypl199c**Δ* mutant, all single gene deletion strains grew as well as the BY4742 wildtype strain in the absence of either FCZ (Figure 4A, 4B) or CS (Figure S1). The *wsc2**Δ* strain exhibited a sensitive phenotype (S) with FCZ treatment (Figure 4A) and a normal growth phenotype (NP) upon treatment with CS (Figure S1). Strains *msa1**Δ*, *gtt1**Δ* and *bmh1**Δ* displayed a sensitive phenotype (S) when treated with FCZ (Figure 4A, Figure 4B, Table S3, and Table S5 respectively) and a normal phenotype (NP) in untreated and CS treated cultures (Figure S1, Table S3, and Table S5). The FCZ sensitivity (S) phenotypes of the single gene deletion strains were all validated by a Colony Forming Unit (CFU) assay (Table S7) (Kwolek-Mirek and Zadrag-Tecza 2014). In contrast, *ypl199c**Δ* cultures displayed slow growth (S*) in the untreated and CS treated cultures, and a normal growth phenotype (NP) in the FCZ treated culture (Figure 4A, Table S3). Thus, the slow growth of the *ypl199c**Δ* mutant was attributed to the *YPL199c* gene deletion with no effect attributed to the stress treatments.

Double mutant strains with *preyΔsensorΔ* combinations were evaluated to determine if any combination displayed a synthetic mutant phenotype. *PreyΔ**wsc2**Δ* double mutants that grew similar to the single strongest mutant phenotype were classified as PPIs that likely participate in a common stress response pathway. Mutant combinations of *preyΔ**wsc2**Δ* that displayed growth phenotypes that were different from their respective single mutant strains were classified as PPIs potentially acting in different stress response pathways. In the absence of added drug, the *egd2**Δ**wsc2**Δ*, *msa1**Δ**wsc2**Δ*, *yck2**Δ**wsc2**Δ*, and *egd2**Δ**wsc3**Δ* double mutants all showed more severe growth inhibition than the corresponding single mutants (Figure 4A, 4B). Inexplicably, the slow growth defect of the *ypl199c**Δ* strain was suppressed in the *ypl199c**Δ**wsc2**Δ* double mutant background.

In the presence of FCZ, the *gtt1**Δ**wsc2**Δ* strain exhibited a sensitive phenotype (Figure 4A, Table S4) as did the individual *wsc2**Δ* and *gtt1**Δ* strains (Figure 4A, Table S3, Table S7). The *yck2**Δ**wsc2**Δ* strain exhibited an FCZ-resistant phenotype (R) (Figure 4A, Table S4) while the individual *yck2**Δ* and *wsc2**Δ* strains displayed (NP) and (S) phenotypes, respectively (Figure 4A, Table S3). As noted above, the *egd2**Δ**wsc2**Δ*, *msa1**Δ**wsc2**Δ*, *yck2**Δ**wsc2**Δ*, *and **egd2**Δ**wsc3**Δ* double mutants all showed some growth inhibition in the absence of FCZ yet each double mutant grew at least as well as the BY4742 control when FCZ was present (Figure 4A, Figure 4B, Table S4, and Table S6 respectively).

Strains bearing *wsc3**Δ* or *bmh1**Δ* single mutations were sensitive (S) to FCZ but no double mutant combinations of *preyΔ**wsc3**Δ* strains showed sensitivity to FCZ or CS (Figure 4A, Figure 4B, Table S5, and Table S6). Curiously, the double mutant strain *egd1**Δ**wsc2**Δ* exhibited normal growth (NP) under all conditions tested. At the same time, the *egd2**Δ**wsc2**Δ* and *egd2**Δ**wsc3**Δ* displayed diminished growth in untreated cultures, and resistant growth (R) in response to FCZ treatment (Figures 4A, Figure 4B, Table S4, and Table S6 respectively).

### Assessment of Wsc2p and Wsc3p interactor function in activation of the CWI pathway

**Figure 5:**
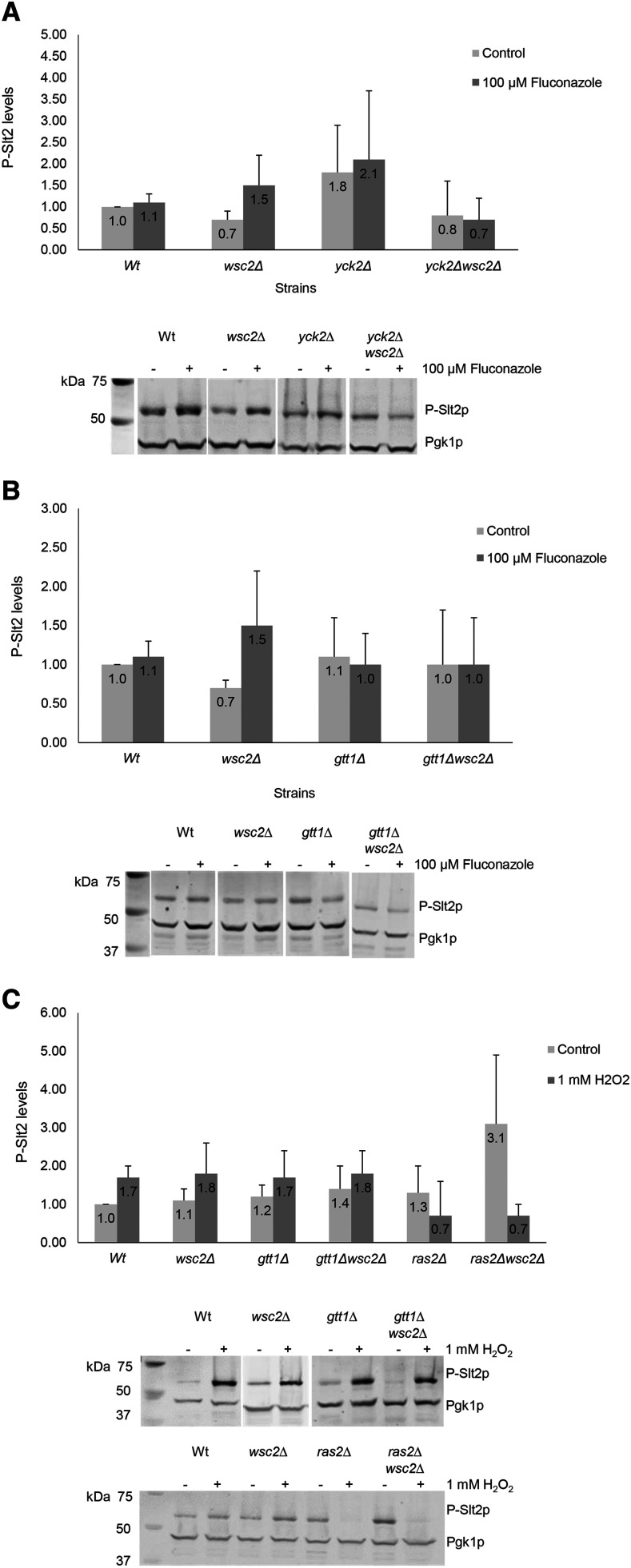
Western blot analysis of phospho-*Slt2p* (P-*Slt2p*) levels in mutant strains of *Wsc2p* and *Wsc3p* and their interactors treated with (A), (B): 100 μM FCZ, and (C) 1 mM H_2_O_2_. Wild type, single, and double mutant cultures were treated with each chemical for 1 hr at 27°. Western blots of total protein extracts derived from treated and control cultures were incubated with anti-phospho p44/42 MAPK (P-*Slt2p*) and anti phosphoglycerate kinase (*Pgk1p*) antibodies as the loading control described in the Materials and Methods. Numerical values shown within each histogram represent fold-change of the P-*Slt2p* intensity in each sample adjusted to the P-*Slt2p* intensity of wild type control (WT) each normalized against *Pgk1p*. Each data point shown represents the mean ± SEM of n ≥ 3.

We wished to determine if the PPIs that acquired a sensitive growth phenotype (S) when mutated were also needed to activate the CWI pathway. To test this, mutant strains containing deletions of genes encoding *Wsc2p* or *Wsc3p* (*sensorΔ*), their interactors (*preyΔ*), or double mutant combinations (*preyΔsensorΔ*) that exhibited a growth defect in the previous analysis were assayed for the accumulation of hyper-phosphorylated *Slt2p* (P-*Slt2p*), the readout for CWI pathway activation. Treatments with antifungal agents CS (Figure S2), FCZ (Figure 5A and 5B), or H_2_O_2_ (Figure 5C) were as described in the previous section. PPIs that exhibited the (S) or (R) growth phenotypes with stress treatment were scored for CWI pathway activation. Strains bearing single mutant *yck2**Δ* and *wsc2**Δ* and the double mutant combination *yck2**Δ**wsc2**Δ*, responded to CS treatment with robust CWI activation, similar to the wild type control (Figure S2).

In contrast to the strong response to CS, wildtype yeast showed little to no activation of the CWI pathway in the presence of 100 µM FCZ. The *wsc2**Δ* single mutant responded to FCZ treatment by weak (<twofold) CWI pathway activation (Figure 5A). However, strains bearing *yck2**Δ* and *yck2**Δ**wsc2**Δ* (Figure 5A), and *gtt1**Δ* and *gtt1**Δ**wsc2**Δ* (Figure 5B), failed to activate the CWI pathway with FCZ addition. Taken together with the results presented above, there does not appear to be a simple consistent correlation between CWI activation and growth phenotype under FCZ stress conditions (Figure 5A, 5B and Figure 4A).

In a previous publication, *Grx1p*, *Atx1p* and *Ras2p*, which represent enzymes involved in the cellular response to oxidative stress, were identified as interactors of the *Wsc1p* and *Mid2p* mechanosensory proteins (Santiago-Cartagena *et al.* 2019). We find that *Gtt1p* a dual function enzyme with glutathione S-transferase and peroxidase activity interacts with *Wsc2p*. Similar to the wildtype, mutant strains bearing *wsc2**Δ*, *gtt1**Δ*, and *gtt1**Δ**wsc2**Δ* displayed normal growth phenotypes (NP) (Figure 4C, Table S3, Table S4) and did not activate the CWI pathway significantly upon treatment with 1mM H_2_O_2_ (Figure 5C). In contrast, the *ras2**Δ* and *ras2**Δ**wsc2**Δ* positive control strains showed the expected sensitive (S) growth in response to H_2_O_2_ treatment (Figure 4C, Table S3 and Table S4) with elevated basal levels of P-*Slt2p* and a diminished capacity for CWI pathway activation as reported previously (Figure 5C) (Park *et al.* 2005; Santiago-Cartagena *et al.* 2019).

### Re-validation of iMYTH protein-protein interactions by Immunoprecipitation-Mass Spectrometry (IP-MS)

**Figure 6:**
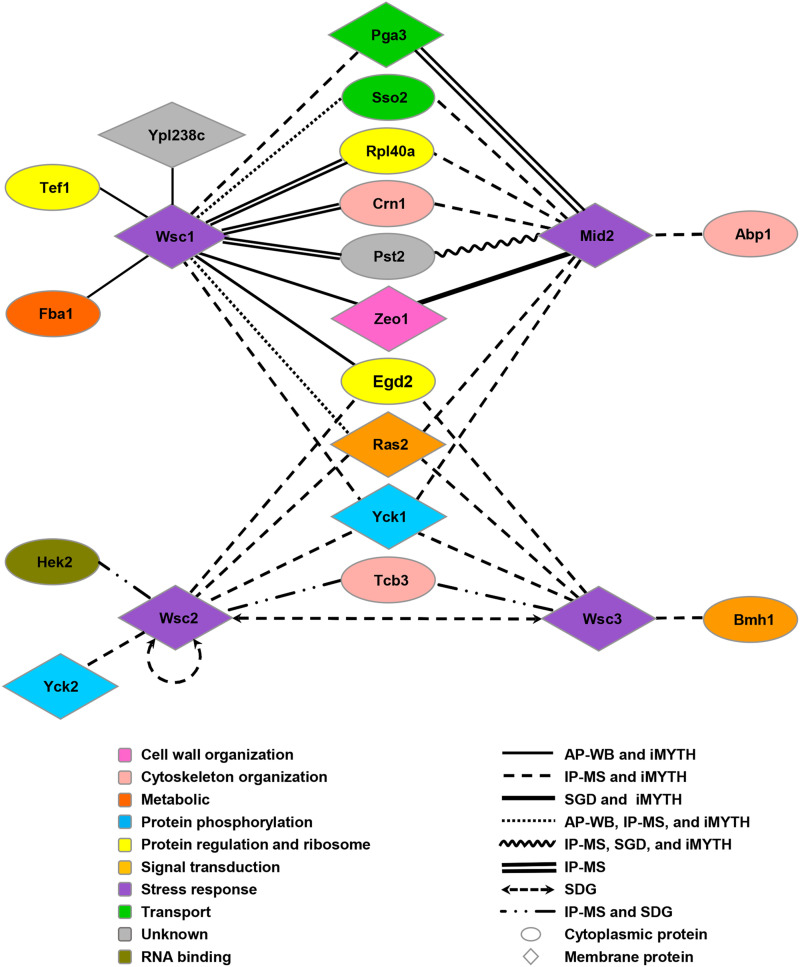
Interactome of *Wsc1p*, *Wsc2p*, *Wsc3p*, and *Mid2p* interactor proteins validated by BDT, AP-WB, and/or IP-MS. Geometric shapes of the nodes represent the cellular localization of the proteins. *Wsc1p*, *Wsc2p*, *Wsc3p*, and *Mid2p* are the bait proteins and the surrounding nodes represent prey proteins. Node fill color-code indicates the biological process for each protein. All edges indicate a physical protein-protein interaction.

To re-validate the results of previous iMYTH BDT confirmations with *Wsc2p* and *Wsc3p* baits, we performed immunoprecipitation (IP) of endogenously expressed GFP-tagged *Wsc2p* and *Wsc3p* baits from total protein extracts and coupled the IPs with Mass Spectrometry (IP-MS) analysis as described previously (Santiago-Cartagena *et al.* 2019). By this method, the iMYTH interactors *Egd2p*, *Yck1p*, *Yck2p*, and *Ras2p* were re-validated as interactors of *Wsc2p* while *Bmh1p*, *Egd2p*, *Yck1p*, and *Ras2p* were re-validated as interactors of *Wsc3p* (Table 2). The IP-MS assay also identified *Hek2p* as an interactor of *Wsc2p*, and *Tcb3p* as an interactor of *Wsc2p* and *Wsc3p*, both of which were previously reported in the SGD PPI database. We have stated previously that protein interactors re-validated by IP-MS are likely to represent proteins with the strongest interactions (Santiago-Cartagena *et al.* 2019). In contrast, iMYTH protein interactors confirmed by the bait dependency test that were not identified in the IP-MS assay may represent transient associations or protein interactions too weak to survive the extraction process. However, we recognize that the introduction of protein tags and other experimental variables may also impact protein recovery. An integrative interactome map with the re-validated IP-MS interactors of *Wsc2p* and *Wsc3p*, as well as our previous data from studies with re-validated interactors of *Wsc1p* and *Mid2p*, their corresponding biological functions, and cellular compartment information is provided (Figure 6).

**Table 2:** Peptides counts and probability percentages for Wsc2p and Wsc3p interactors identified by iMYTH and validated by GFP immunoprecipitation coupled to Mass Spectrometry analysis (IP-MS)

Protein Name	WT*^a^*			Wsc2*^a^*			Wsc3*^a^*		
	TP	TP	TP	TP	TP	TP	TP	TP	TP
Wsc2	0	0	0	156(99.58)	87(99.58)	12(99.58)	0	0	0
Wsc3	0	0	0	0	0	0	18(99.58)	6(99.58)	17(99.58)
Bmh1	0	0	0	0	0	0	5(99.58)	0	0
Egd2	0	0	1(59.22)	4(99.52)	2(99.52)	2(96.06)	3(99.58)	3(99.45)	0
Yck2	0	0	0	3(99.58)	2(99.58)	2(99.58)	2(99.58)	0	0
Hek2	0	0	0	2(99.58)	3(99.58)	0	0	0	0
Tcb3	0	0	0	6(99.58)	2(99.58)	2(99.58)	2(99.58)	1(97.33)	1(99.58)
Yck1	0	0	0	3(99.58)	2(99.58)	4(99.58)	1(99.58)	3(99.58)	5(99.58)
Yck2	0	0	0	2(99.58)	2(99.58)	2(99.58)			
Ras2*^b^*	0	0	0	6(99.58)			7(99.58)		
Yck1*^b^*	0	0	0	0			2(99.58)		

a TP = Total peptides, (Probability percentage), N = 3.

b TP = Total peptides, (Probability percentage), N = 1.

### Bioinformatics analysis for identification of homologous proteins in other fungal species

Bioinformatics analysis was performed using the application MUSCLE (Edgar R.C., 2004) to identify protein sequences with homology to *Wsc2p* and *Wsc3p* and the 12 iMYTH interactors. Homologous proteins for *Wsc2p*, *Wsc3p* and their interactors were identified in *Aspergillus fumigatus*, *Candida albicans*, *Cryptococcus neoformans*, and *Homo sapiens* (Table 3). Homologous sequences for all except *Gtt1p*, *Zeo1p*, and *Wsc1p* were identified in humans making this fungal specific set of proteins potentially valuable therapeutic targets.

**Table 3:** Quantity of homologous protein sequences for each interactor and sensor protein by organism

	Organisms				
Interactor or Sensor Protein Name	*Yeast (Query Sequence)*	*Aspergillus fumigatus*	*Candida albicans*	*Cryptococcus neoformans*	*Homo sapiens*
Bmh1	BMH1	2	2	3	2
		Y699_03118	BMH1	CNBI2830	YWHAE
		Y699_03834	BMH1	CNAG_05235	HEL2
				CNL03930	
Cpr1	CPR1			11	
				CNAG_03627	
				CPA1	
		3		CNB01290	
		Y699_01807	2	CPA1	3
		asp f 27	CYP1	CPA2	PPIF
		Y699_06153	CYP1	CNAG_03621	PPIF
				CNAG_03621	N/A
				CNBB4490	
				CNB01230	
				CPA2	
				CNBB4430	
Egd1		1	2	3	3
	EGD1	Y699_07424	EGD1	CNAG_05437	BTF3L4
			EGD1	EGD1	BTF3L4
				EGD1	BTF3
Egd2	EGD2			0	6
					hCG_2016482
			2		NACA
		1	EGD2		NACA
		Y699_07319	EGD2		NACA
					NACAP1
					NACAD
Gtt1	GTT1	3	4	0	0
		Y699_09395	GTT12		
		Y699_02150	GTT11		
		Y699_05729	GTT13		
			GTT1		
Msa1	MSA1	0	0	0	2
					ZNF384
					ZNF384
Ras2	RAS2			13	
				CNAG_04119	
				CNBH4090	
				CNI04280	8
				RAS1	NRAS
		3	3	CNAG_00293	NRAS
		Y699_00700	ROM2	RAS1	HRAS
		Y699_00948	RAS1	RAS1	KRAS
		Y699_00594	RAS1	CNB01780	KRAS
				CNAG_03680	RRAS
				CNBM1020	KRAS
				CNM01160	KRAS
				CNAG_07903	
				CNBB3940	
Yck1	YCK1	1 Y699_03846			9
					CSNK1G3
			3	5	CSNK1G2
			CAALFM_C208270CA	CNBA5210	CSNK1G3
			YCK2	CNBA5210	CSNK1G3
			HRR25	CNA05390	CSNK1G3
				CNAG_00556	hCG_2004507
				CNAG_00556	CSNK1G1
					CSNK1G1
					CSNK1G2
Yck2	YCK2	1 Y699_03846	2 CAALFM_C208270CA YCK2	6	5
				CNBA5210	CSNK1G3
				CNBA5210	CSNK1G3
				CNAG_00556	CSNK1G3
				CNA05390	CSNK1G2
				CNA05390	CSNK1G3
				CNAG_00556	
Ypl199c	YPL199C	1 Y699_05052	1 Y699_05052	3	3
				CNBF2620	N4BP2
				CNF02090	N4BP2
				CNAG_05769	N4BP2
Zeo1	ZEO1	0	1 CTA2	0	0
Mid2	MID2	0	2 orf19.4906 DFI1	6	
				C365_06939	
				CNAG_05550	2
				C356_06923	TPRXL
				CNH01980	hCG_2042888
				CNH01980	
				CNH01980	
Mtl1	MTL1	0		0	8
					TPRXL
			3		hCG_2042888
			orf19.4906		MUC5AC
			PGA55		MUC21
			RBR3		MUC21
					MUC21
					MUC21
					FLJ50027
Wsc1	SLG1	1 CENPK1137D_2030		13	0
				CNAG_05550	
				CNH01980	
			6	CNBL1980	
			WSC2	CNH01980	
			WSC1	CNAG_00668	
			CHT3	CNBD2600	
			CHT3	CND03720	
			CAALFM_C110350CA	CNAG_03328	
			WSC4	CNBC2510	
				CNC04670	
				CNAG_00475	
				CNBG3600	
				CNG01200	
Wsc2	WSC2	1 Y699_05984		8	
			5	CNAG_07659	
			WSC2	CNH01980	3
			CAALFM_C110350CA	CNBL1980	MUC5AC
			CHT3	CNAG_05550	hCG_2042888
			WSC4	CNAG_03328	TPRXL
			WSC1	CNBG2260	
				CNH01980	
				CNG02510	
Wsc3	WSC3	1 Y699_09176	4		
			WSC2	3	3
			CAALFM_C110350CA	CNAG_05550	MUC5AC
			CHT3	CNH01980	TPRXL
			CHT3	CNBL1980	hCG_2042888

## Discussion

This study describes 12 novel protein-protein interactions (PPIs) identified by iMYTH screening, collectively referred to as “interactors” of the mechanosensors *Wsc2p* and *Wsc3p* of *S. cerevisiae*. With the exception of *Zeo1p*, all the iMYTH interactors of *Wsc2p* and *Wsc3p* that we identified were novel. A protein interaction network or “interactome” was assembled using the validated PPIs identified by iMYTH (Figure 3). A second interactome image presents the seven iMYTH interactors that scored positive in secondary IP-MS analysis combined with previously defined iMYTH interactors of *Wsc1p* and *Mid2p* (Figure 6) (Santiago-Cartagena *et al.* 2019). This display reveals multiple redundant PPIs among these mechanosensory proteins. Interestingly, among the iMYTH interactors of *Wsc2p* and *Wsc3p* that were re-validated by IP-MS, only *Bmh1p* exhibited a growth defect in functional tests with FCZ. The interactors *Gtt1p* and *Msa1p* which also exhibited growth defects with FCZ were not re-validated by IP-MS. Therefore, we suggest that although re-validation of iMYTH results by alternative methods such as IP-MS provides important information about the nature of these PPIs, this criterion alone should not be used to exclude PPIs of potential interest.

Expression of *Wsc2p* appeared to be more relevant for stress resistance than *Wsc3p* if judged by the sensitive responses of three of its unique interactors (*Ras2p*, *Yck2p*, and *Gtt1p*) in functional tests with the potent antifungal drug FCZ and the strong oxidant H_2_O_2_. Specifically, *gtt1**∆* and *wsc2**∆* in both the single or double mutant combinations exhibited sensitivity to treatments with FCZ. These results suggest that *Gtt1p* and *Wsc2p* have an additive effect to promote resistance to FCZ treatment. Yeast bearing *egd2**∆* or *yck2**∆* in combination with *wsc2**∆*, exhibited a resistant growth phenotype (R) with FCZ treatment. The *egd2**∆**wsc3**∆* double mutant responded similarly. These results indicate that both *Egd2p*, *Yck2p* down-regulate growth when membrane stress is induced by FCZ treatment. Deletion of *EGD2* and *YCK2* in the *wsc2**∆* background therefore reverses the sensitivity to FCZ in *wsc2**∆* and *wsc3**∆* mutants. We speculate that the absence of *Egd2p* in the nascent polypeptide associated complex (NAC), which results in diminished translational capacity (Kirstein-Miles *et al.* 2013), enhances fitness in the *wsc2**Δ* background when membrane biogenesis is impaired by FCZ. What links the control of translational capacity to drug resistance is not known. A possible role for *Yck2p* as the phosphorylating kinase of *Wsc2p*, proposed to be a negative regulatory step in CWI activation, is discussed below.

The *Gtt1p* interactor of *Wsc2p* is a plasma membrane localized enzyme with bifunctional glutathione S-transferase and peroxidase activities. Conceivably, the loss of *Gtt1p* activity during H_2_O_2_ treatment might impair growth through oxidative damage to important cellular proteins (Hawkins and Davies 2019). However, the absence of either *Gtt1p* and/or *Wsc2p* had little or no impact on growth or CWI pathway activation in response to H_2_O_2_ treatment, although both proved important for efficient growth in response to FCZ treatment implicating them in a specific role for FCZ resistance.

Based on the prevailing model, the phosphorylation of serine residues within the regulatory C-terminal region of the Wsc1 mechanosensory protein inhibits *Rom2p* binding (Vay *et al.* 2004). The shared *Yck1p* and *Yck2p* interactors are serine-threonine protein kinases. These protein kinases may phosphorylate this serine-rich motif, specifically S319, S320, S322, S323, at the *Wsc1p* C-terminus (Vay *et al.* 2004) and equivalent positions within the *Wsc2p*, *Wsc3p* and *Mid2p* mechanosensory proteins that likewise interact with *Yck1p* or *Yck2p*. In such a case, the absence of these phosphorylation events would be predicted to enhance CWI activation (viewed as elevated P-*Slt2p*) under stress conditions. While the *yck2**Δ* strain displayed a trend toward elevated basal levels of P-*Slt2p* in both the FCZ treated and untreated cultures, which is consistent with this prediction (Figure 5A), this strain exhibited slow growth (S) under FCZ treatment (Figure 4A). In contrast, the *yck2**Δ**wsc2**Δ* strain displayed suppressed levels of CWI activation (Figure 5A) yet this strain exhibited resistant growth (R) under FCZ treatment (Figure 4A). These observations indicate that independent of its putative negative regulatory role in CWI activation, *Yck2p* expression is required for normal growth under FCZ treatment conditions (exhibited by the slow growth of a *yck2**Δ* strain, Figure 4A), while it is dispensable under the same conditions when *Wsc2p* is absent (exhibited by the resistant growth of a *yck2**Δ**wsc2**Δ* strain, Figure 4A). We propose that in a BY4742 control strain under normal culture conditions, *Yck2p* is capable of suppressing *Wsc2p* interaction with *Rom2p* through phosphorylation of the *Wsc2p* C-terminus (maintaining normal growth and preventing premature CWI activation). Under FCZ treatment conditions, *Wsc2p* may be released from this suppression state either by a deletion of *Yck2p* in *yck2**Δ* strains, or by a proposed enzymatic dephosphorylation of its C-terminus (maintaining normal growth equivalent to BY4742 and elevated CWI activation). The absence of *Yck2p* in the *yck2**Δ* mutant therefore elevates the baseline level of P-*Slt2p*, a phenotype that resembles elevated steady state CWI activation in *ras2**Δ* mutants (Park *et al.* 2005, Santiago-Cartagena *et al.* 2019). The absence of both *Wsc2p* and *Yck2p* in a double mutant *yck2**Δ**wsc2**Δ* allows the strain to maintain Resistant growth (R) (Figure 4A) but with a complete loss in its capacity to activate the CWI pathway (Figure 5A). This is consistent with the idea that expression of both *Wsc2p* and *Yck2p* is required to maintain the capacity to activate the CWI pathway under this type of stress. A stress inducible phosphatase *Sdp1p* has been proposed to be responsible for de-phosphorylation of the C-terminal serine residues of *Wsc1p* (Hahn and Thiele 2002). Given that *Yck2p* and *Yck1p* are physical interactors of *Wsc2p* and *Wsc3p*, the interplay between these putative phosphorylating kinases and the phosphatase *Sdp1p* should be revisited in the context of stress resistance and CWI pathway induction.

The *Hek2p* RNA binding protein was previously identified by Hasegawa *et al.* (2008) as a physical interactor of *Wsc2p* as illustrated in Figure 6. Interestingly, *Hek2p* is reported to represses translation of *ASH1* mRNA while *Yck1p*-dependent phosphorylation of the RNA-binding protein Khd1p activates *ASH1* mRNA translation. While *Khd1p* is not an interactor of *Wsc2p*, the PPIs of *Yck1p* and *Hek2p* with *Wsc2p* raises the possibility that their association with *Wsc2p* at the plasma membrane may be necessary for regulating *ASH1* mRNA translation in daughter cells.

*Ras2p* is a small GTPase that regulates adenylate cyclase activity and cAMP synthesis (Conrad *et al.* 2014). The yeast *Ras2p* shares approximately 65% of amino acid sequence homology with the conserved human KRAS, HRAS, and RRAS proteins (Table 3). An interaction reported previously between *ras2**∆* and *rom2**∆* mutants in yeast suggested that *Ras2p* expression modulates *Rom2p*-dependent activation of the CWI pathway (Park *et al.* 2005). In the same study, while the *ras2**∆* mutant accumulated elevated baseline levels of phospho-*Slt2p* (corroborated in our studies), it down regulated MAPK activation in response to heat shock. Overexpression of *Rom2p* did not overcome the down regulation of CWI in this *ras2**∆* mutant. In our current study, *Ras2p* was confirmed as an interacting partner of *Wsc2p* and shown to have a pivotal role in response to oxidative stress induced by H_2_O_2_, consistent with other reports (Vilella *et al.* 2005). We find that *ras2**∆* mutants exhibited elevated baseline levels of phospho-*Slt2p* in untreated cells and fail to accumulate additional phospho-*Slt2p* following oxidative stress treatment. An explanation for how the P-*Slt2p* levels drop in the *ras2**Δ* mutants during H_2_O_2_ treatment, rather than remaining stable at the untreated baseline levels, may be that H_2_O_2_ shuts down the CWI pathway almost completely in these strains. Normal turnover of the protein would then produce a drop in P-*Slt2p* steady state levels to below untreated control levels.

The physical interaction of *Ras2p* with *Wsc2p* and *Wsc3p* reported here supports the hypothesis that *Ras2p* may modulate binding of *Rom2p* at the C-terminus of multiple Wsc-family mechanosensory proteins simultaneously. Going forward, it will be important to determine if *Ras2p* and *Rom2p* directly bind to the C-terminal sequence of *Wsc2p* (and perhaps other mechanosensors) or interact via common linker proteins.

Multiple strains such as *ypl199c**Δ*, *msa1**Δ**wsc2**Δ*, *yck2**Δ**wsc2**Δ*, and *egd2**Δ**wsc3**Δ* (Figure 4A and 4B, Tables S3, S4, and S6) exhibited synthetic growth defects under control conditions that were not changed by the addition of the CS or FCZ chemical stressors. While the underlying basis of these genetic interactions is unknown, presumably they relate to the functions of the encoded proteins outside the context of CWI pathway response. The *YPL199c* interactor encodes a protein of unknown function that is predicted to be palmitoylated (Ren *et al.* 2008). This study contributes a novel descriptor for the *Ypl199c* protein as a physical interactor of *Wsc2p*, consistent with its proposed membrane association. *Bmh1p* was the only interactor of *Wsc3p* other that *Wsc3p* itself that exhibited sensitivity to FCZ treatment (Figure 4B, Table S5). *Bmh1p* is described as a 14-3-3 protein that has been linked to Ras/MAPK and rapamycin-sensitive signaling (Gelperin *et al.* 1995). While the relationship of *Bmh1p* to *Ras2p* was not specifically addressed by our study, *Bmh1p* and *Ras2p* association with *Wsc3p* provide intriguing possibilities for integration of the CWI and TOR signaling pathways.

To evaluate the translational potential of the PPIs identified in *S. cerevisiae* with other fungal organisms of medical importance, the amino acid sequences of 12 interactor proteins of *Wsc2p* and *Wsc3p* were evaluated for likely orthologs in *Candida albicans*, *Aspergillus fumigatus*, *Cryptococcus neoformans*, and humans. As expected, virtually all of these proteins, except for *Wsc1p*, *Gtt1p* and *Zeo1p*, are conserved across multiple species of fungi and in humans (Table 3). These observations support broad phylogenetic conservation of the CWI signaling (Lam *et al.* 2013; Dichtl *et al.* 2016; Rosenwald *et al.* 2016). However, the absence of apparent *Wsc1p*, *Gtt1p*, and *Zeo1p* homologs in humans, suggests that these proteins represent potentially valuable therapeutic targets in the fight against fungal infections.
